# A distributed active patch antenna model of a Josephson oscillator

**DOI:** 10.3762/bjnano.14.16

**Published:** 2023-01-26

**Authors:** Vladimir M Krasnov

**Affiliations:** 1 Department of Physics, Stockholm University, AlbaNova University Center, SE-10691 Stockholm, Sweden

**Keywords:** antenna theory, cavity modes, Josephson effect, terahertz radiation

## Abstract

Optimization of Josephson oscillators requires a quantitative understanding of their microwave properties. A Josephson junction has a geometry similar to a microstrip patch antenna. However, it is biased by a dc current distributed over the whole area of the junction. The oscillating electric field is generated internally via the ac-Josephson effect. In this work, I present a distributed, active patch antenna model of a Josephson oscillator. It takes into account the internal Josephson electrodynamics and allows for the determination of the effective input resistance, which couples the Josephson current to cavity modes in the transmission line formed by the junction. The model provides full characterization of Josephson oscillators and explains the origin of the low radiative power efficiency. Finally, I discuss the design of an optimized Josephson patch oscillator capable of reaching high efficiency and radiation power for emission into free space.

## Introduction

A flux-flow oscillator (FFO) is the most extensively studied Josephson source of high-frequency electromagnetic waves (EMW) [1–12]. A FFO was used in the first direct demonstration of Josephson emission by Yanson et al., back in 1965 [13–14]. State of the art FFOs, developed by Koshelets and co-workers show a remarkable performance in terms of tunability and linewidth [6,9,12]. However, they emit very little power into free space [11,13,15–16]. The low radiation power efficiency, that is, the ratio of radiated to dissipated power, is commonly attributed to a large impedance mismatch between a Josephson junction (JJ) and free space [10,16–17]. But there is no consensus about the value of the junction impedance: Is it very small [16] or, in contrast, very large [10]? At present, there is no clear understanding about what causes the impedance mismatch and which geometrical parameters should be changed for solving the problem. The discovery of significant terahertz emission from stacked intrinsic JJs in layered high-*T*_c_ cuprates [18–27] further emphasizes the necessity of a quantitative understanding of microwave emission from Josephson oscillators.

**Figure 1:**
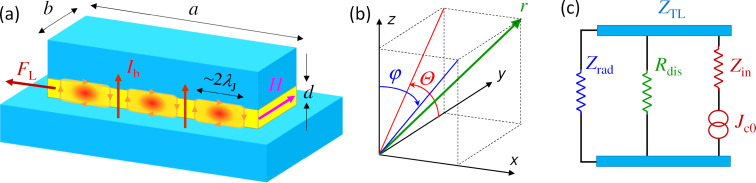
(a) A sketch of the Josephson flux-flow oscillator. It is based on a sandwich-type junction with two superconducting electrodes (light blue) separated by a dielectric interlayer (yellow). Red ovals represent Josephson vortices that are driven by the Lorentz force, *F*_L_, exerted by the dc bias current, *I*_b_. From the outside, the junction has a patch antenna geometry. However, inside it is driven by a distributed dc current, and the oscillating voltage is generated internally by a combination of the ac-Josephson effect and the flux-flow phenomenon. (b) Clarification of spatial and angular coordinates. (c) An equivalent circuit of the Josephson junction. The ac-Josephson effect provides a source of the high-frequency alternating current with the fixed amplitude of current density, *J*_c0_. The oscillating voltage at the junction edges is generated by means of the input junction impedance, *Z*_in_, and is distributed between the internal dissipative resistance, *R*_dis_, and the external radiative impedence, *Z*_rad_, connected by the transmission line impedance *Z*_TL_.

Figure 1a shows a sketch of a typical FFO. It is based on a sandwich-type (overlap) JJ with the length, *a* ≫ λ_J_, much larger than the Josephson penetration depth, and both in-plane sizes much larger than the thickness of the junction interface, *d* ≪ *b* ≪ *a*. The in-plane magnetic field, *H_y_*, introduces a chain of Josephson vortices (fluxons) in the JJ. The dc bias current, *I*_b_, exerts a Lorentz force, *F*_L_, and causes a unidirectional fluxon motion. Upon collision with the junction edge, the fluxons annihilate. The released energy produces an EMW pulse, which is partially emitted but mostly reflected backwards in the JJ. Propagation and reflection of FFO pulses in the transmission line (TL) formed by the JJ leads to the formation of standing waves. The corresponding cavity mode resonances are manifested by Fiske steps in the current–voltage (*I*–*V*) characteristics [16,28–32]. FFOs exhibit sharp emission maxima at the Fiske steps [9,12–13]. Such a conditional emission indicates that several additional and equally important phenomena (apart from the ac-Josephson effect) are involved in FFO operation [10]. The excitation of high-quality factor, *Q* ≫ 1, cavity modes is one of them.

Geometry is playing a decisive role for characteristics of microwave devices. Although calculations of radiative impedances of JJs do exist [33], they were not made for the FFO geometry. From the outside, the overlap JJ looks like a well-known microstrip patch antenna [34–36]. The difference, however, is inside. A standard patch antenna has a point-like feed-in port, while in a JJ the bias current is distributed over the whole area of the JJ. Furthermore, the oscillating component of the current is actively generated inside the JJ by means of the ac-Josephson effect and the flux-flow phenomenon. Therefore, a JJ can be considered as an actively pumped patch antenna with a distributed feed-in current.

In this work, I present a distributed, active patch antenna model of a Josephson oscillator. It expands the TL model of a patch antenna [36], taking into account the spatial distribution of the input current density in a JJ, described by the perturbed sine-Gordon equation. In the presence of a magnetic field and fluxons, the oscillating current is distributed nonuniformly within the junction. This nonuniformity is essential for the FFO operation. It determines the variable input resistance, which enables the coupling of the Josephson current to cavity mode resonances in the junction. The presented model allows for the application of many of patch antenna results and facilitates full characterization of Josephson oscillators, including the emission power, directivity, and power efficiency. The model explains the origin of the low power efficiency for emission in free space and clarifies which parameters can be changed to improve the FFO characteristics. Finally, I discuss the design of a Josephson patch oscillator that can reach high power for emission in free space with the optimal power efficiency of approx. 50%.

## Results

The spatial-temporal distribution of voltage in a JJ is described by the equation (see chapter 9 in [31]):


[1]

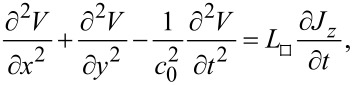



where *c*_0_ is the (Swihart) velocity of EMWs in the TL formed by the JJ and *L*_□_ is the inductance of JJ per square. *J_z_* is the current density through the JJ, which has Cooper pair and quasiparticle (QP) components,


[2]

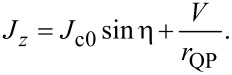



Here, *J*_c0_ is the Josephson critical current density, η is the Josephson phase difference, and *r*_QP_ = *R*_QP_*ab* is the QP resistance per unit area.

### Active patch antenna model of a junction

Equation 1 is the equation for an active TL [37] with a distributed feed-in current density *J_z_*. Therefore, a JJ has many similarities with the microstrip patch antenna. However, there are three main differences:

(i) The feed-in geometry. A patch antenna has a point-like feed-in port, through which the oscillating current is applied [34–36]. The FFO is biased by a dc current distributed over the whole JJ area.

(ii) The excitation scheme. A patch antenna is a linear oscillator pumped by a harmonic signal. In contrast, a JJ is biased by a dc-current and the oscillatory component is generated inside the JJ via the ac-Josephson effect and the flux-flow phenomenon.

(iii) The slow propagation speed of EMWs inside the JJ, *c*_0_ ≪ *c*. This is caused by a large kinetic inductance of superconducting electrodes. For Nb-based JJs, *c*/*c*_0_ ≈ 40 (see the estimation in section Discussion). For atomic-scale intrinsic JJs in layered cuprates, *c*_0_ can be almost 1000 times slower than *c* [32]. Because of that, the wavelength inside the JJ is much smaller than in free space, λ ≪ λ_0_. Therefore, a JJ corresponds to a patch antenna with an extraordinary large effective permittivity, 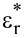 = (*c*/*c*_0_)^2^.

The dynamics of a JJ is described by a nonlinear perturbed sine-Gordon equation,


[3]

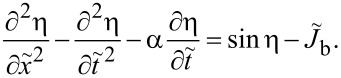



It follows from Equation 1 and Equation 2, taking into account the ac-Josephson relation, *V* = (Φ_0_/2π)∂η/∂*t*. Equation 3 is written in a dimensionless form with space, 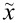 = *x*/λ_J_, normalized by λ_J_,and time, 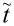 = ω_p_*t*, by the Josephson plasma frequency, ω_p_. Here α is the QP damping factor, and 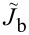 = *J*_b_/*J*_c0_ is the normalized bias current density, which originates from the ∂^2^*V*/∂*y*^2^ term in Equation 1 [38]. In what follows, “tilde” will indicate dimensionless variables, 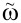 = ω/ω_p_ and 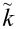 = λ_J_*k*. The definition of and the interconnection between different variables are clarified in the Appendix section.

### Radiative resistance of a patch antenna

A rectangular patch antenna has two radiating slots, which correspond to the left and right edges of the JJ in Figure 1a. The slots can be considered as magnetic current lines (magnetic dipoles) [39]. Radiation from the antenna is determined by the radiative impedance, *Z*rad. For a patch with a very thin insulator (as is the case for a tunnel JJ), the radiative admittance of one slot, 1/*Z*_rad1_ = *G*_1_ + *iB*_1_, contains a large imaginary part *B*_1_, caused by the large capacitance. However, at the cavity mode resonance the imaginary contributions from the two slots cancel out [34,36,39] and the radiative impedance becomes real. Therefore, at the resonance the radiation power from one slot is


[4]

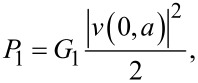



where |*v*(0,*a*)| is the amplitude of voltage oscillations at the slot (*x* = 0,*a*) and *G*_1_ is the radiative conductance of the single slot. Low-*T*_c_ JJs are operating at sub-terahertz frequencies, for which the wavelength in free space is large, λ_0_ ≫ *b* ≫ *d*. In this limit [36,39],


[5]

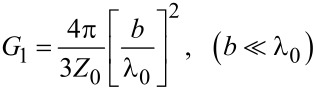



where *Z*_0_ = 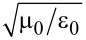 ≃ 376.73 (Ω) is the impedance of free space.

To calculate the total radiation power from both slots one has to take into account the mutual radiative conductance, *G*_12_, and the array factor AF [36]. *G*_12_ is originating from a cross product of electric and magnetic fields generated by different slots. For λ_0_ ≫ *b* ≫ *d* it is equal to [36,40]


[6]

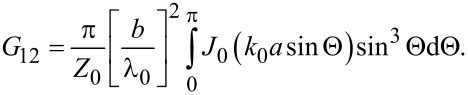



Here, *J*_0_ is the zeroth-order Bessel function, *k*_0_ = 2π/λ_0_ is the wave number in free space, and the angle Θ is defined in Figure 1b. For the *n*-th cavity mode,


[7]

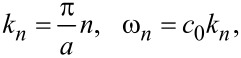



the argument of *J*_0_ becomes (*c*_0_/*c*)π*n*sinΘ. Since *c*_0_ ≪ *c*, *k*_0_*a* is small. Expanding in Equation 6, *J*_0_(*x*) ≃ 1 − *x*^2^/4 (for *x* ≪ 1), we obtain:


[8]

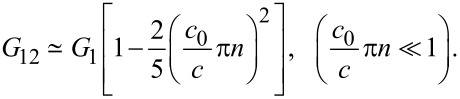



It is seen that the mutual conductance for a JJ with thin electrodes (slow *c*_0_) is not negligible and can be as big as the single-slot conductance *G*_1_, Equation 5.

The array factor takes into account the interference of electromagnetic fields from the two slots in the far field. It depends on the separation between the slots, *a*, the relative phase shift, β, and the direction (φ,Θ). Since radiation from a patch antenna is induced by magnetic current lines, it is more intuitive to consider the interference of magnetic fields, *H*_1_ + *H*_2_ = AF*H*_1_. For the geometry of Figure 1a and Figure 1b, it can be written as [36,40]


[9]

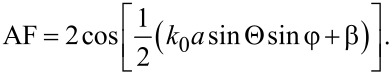



Odd-number cavity modes have antisymmetric voltage oscillations but symmetric magnetic currents, β = 0. This leads to a constructive interference with the maximum AF = 2 perpendicular to the patch along the *z*-axis. For even modes its vice versa, β = π, and a destructive interference leads to a node, AF = 0, along the *z*-axis.

The total emission power is


[10]

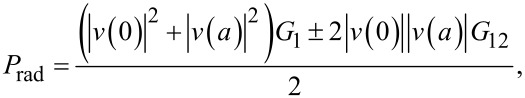



where the plus/minus signs are for odd/even modes, respectively. For equal amplitudes, |*v*(0)| = |*v*(*a*)|,


[11]

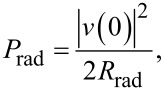



with the effective radiative resistance


[12]

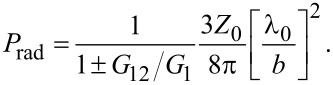



### Determination of voltage amplitudes

To calculate *P*_rad_, we need voltage amplitudes at the JJ edges. Within the TL model of patch antennas, *v*(*x*) is obtained by decomposition into a sum of cavity eigenmodes [34]. For JJs, a similar approach is used for the analysis of Fiske steps [16,29–31]. To separate dc and ac components, we write


[13]

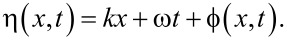



Here, *k* = 2π(Φ/Φ_0_)/*a* is the phase gradient induced by the external field, where Φ is the flux in the JJ. ω = 2πΦ_0_*V*_dc_ is the angular Josephson frequency proportional to the dc voltage *V*_dc_. The last term, ϕ, represents the oscillatory component induced by cavity modes and fluxons. This term generates the ac voltage, which we aim to determine:


[14]

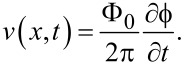



#### Small-amplitude, multimode analysis

In the small-amplitude limit, ϕ ≪ 1, a perturbation approach can be used. A linear expansion of Equation 3 yields [16,29,31],


[15]

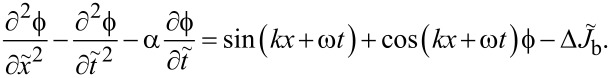



Here, 
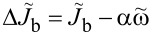
 is the excess dc current with respect to the ohmic QP line. It is caused by the second term on the right-hand side, which enables nonlinear rectification of the Josephson current. The excess dc current is defined as


[16]

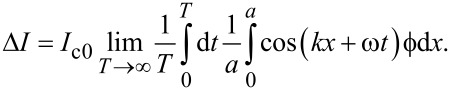



The oscillatory part is described by the equation


[17]

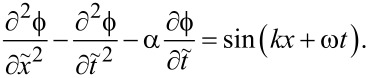



A comparison with Equation 1 shows that this is the active TL equation in which the supercurrent wave, sin(*kx* + ω*t*), acts as a distributed (*x*,*t*)-dependent drive.

To obtain ϕ, a decomposition into cavity eigenmodes is made [15–16,29,31], similar to the TL analysis of patch antennas [34–36]:


[18]

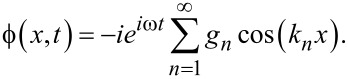



Note that Equation 18 does not include the dc term, *n* = 0, which is accounted for in 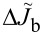 instead, so that ϕ generates solely ac voltage, as described by Equation 14. Substituting Equation 18 in Equation 17 and taking into account the orthogonality of eigenfunctions, one obtains


[19]

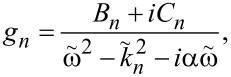




[20]

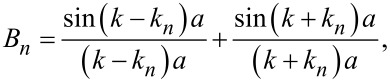




[21]

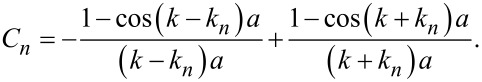



From Equation 14, voltage amplitudes at radiating slots are:


[22]

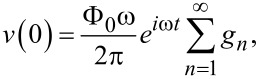




[23]

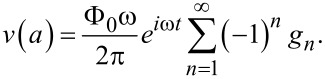



#### Excess current

Without geometrical resonances, the dc current, well above the field-dependent critical current, *I* ≫ *I*_c_(*H*), is determined by the QP resistance, *I* = *V*/*R*_QP_. In dimensionless units, *I*/*I*_c0_ = α*V*/*V*_p_, where *V*_p_ = Φ_0_ω_p_/2π is the voltage at plasma frequency. At resonances, a partial rectification of the oscillating supercurrent occurs, leading to the appearance of Fiske steps in the *I*–*V* curves. The excess dc current, obtained from Equation 16, is [16,29,31]


[24]

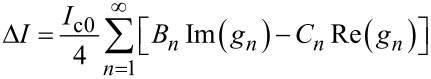



**Figure 2:**
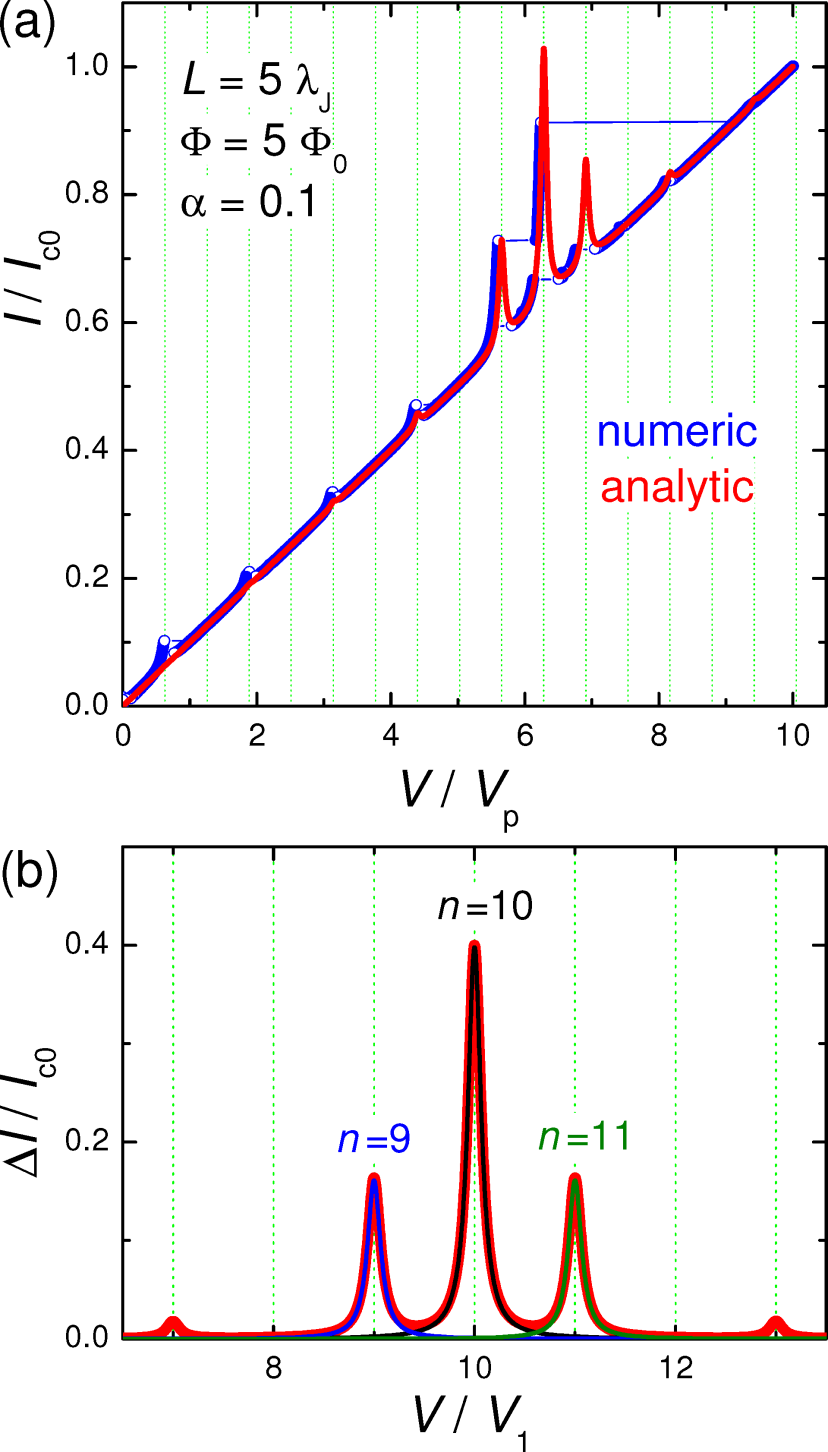
(a) Simulated current–voltage characteristics of a junction with *L* = 5λ_J_, Φ/Φ_0_ = 5 and α = 0.1. Blue symbols represent the full numeric solution of the sine-Gordon equation (up and down current sweep). The red line represents the approximate (perturbative) analytic solution, *I* = *V*/*R*_QP_ + Δ*I*. (b) Excess dc current, Δ*I*(*V*), at Fiske steps. The thick red line represents the multimode analytic solution, Equation 24. Thin blue, black, and olive lines show single-mode solutions for *n* = 9, 10, and 11, respectively. Vertical grid lines in (a) and (b) mark Fiske step voltages. Voltages are normalized by (a) the plasma frequency voltage, *V*_p_, and (b) the lowest Fiske step voltage, *V*_1_.

Figure 2a shows calculated *I*–*V* characteristics of a JJ with *a* = 5λ_J_, α = 0.1 and at a magnetic field corresponding to Φ = 5Φ_0_ in the JJ. Blue symbols represent the direct numerical simulation of the sine-Gordon Equation 3 for up and down current sweep. The red line shows the analytic solution with the excess current given by Equation 24. The agreement between exact (without linearization) numeric and (approximate) analytic solutions is quite good. It is seen that a series of Fiske steps appear in the *I*–*V*. Vertical grid lines mark positions of cavity mode resonances, ω/*c*_0_ = *k_n_*. Fiske steps appear at this condition because of the vanishing of 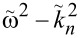 term in the denominator of *g_n_*, Equation 19. The main step occurs at the double resonance condition, ω/*c*_0_ = *k_n_* = *k*. It happens at *n* = 2Φ/Φ_0_ and leads to the vanishing of (*k* − *k_n_*) in the denominators of Equation 20 and Equation 21. The condition, ω/*c*_0_ = *k*, is referred to as the velocity matching because at this point the velocity of the fluxon chain (or phase velocity of the current wave in Equation 17) reaches *c*_0_ [16].

#### Single-mode analysis

Figure 2b shows the excess current, Δ*I*/*I*_c0_ versus *V*, normalized by the *n* = 1 Fiske step voltage, *V*_1_ = Φ_0_*c*_0_/2*a*. Such normalization clearly shows that the main resonance occurs at *n* = 2Φ/Φ_0_ = 10. The thick red line represents the full multimode solution, Equation 24. Thin blue, black, and olive lines represent a single eigenmode contribution for *n* = 9, 10, and 11, respectively. A perfect coincidence with the red line indicates that for underdamped JJs, α ≪ 1, it is sufficient to consider just a single mode. This greatly simplifies the analysis.

For a resonance at mode *n*,


[25]

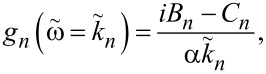



and


[26]

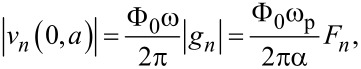




[27]

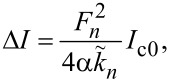



where


[28]

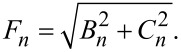



#### Large-amplitude case

The described above perturbative approach is valid only for small amplitudes. Simulations in Figure 2a are made for an underdamped JJ, α = 0.1. In this case the quality factor of high-order cavity modes is large,




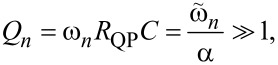




and |*g_n_*| is not small. Since ϕ appears within the sin η term in Equation 3, the maximum possible amplitude of |*g_n_*| is π. This reflects one of the key differences between FFO and patch antenna. The patch antenna is a linear element in which the voltage amplitude is directly proportional to the feed current. A FFO is essentially nonlinear. The amplitude of Josephson phase oscillations will not grow beyond |*g_n_*| = π. Instead, higher harmonic generation will occur.

Full numerical simulations of the sine-Gordon equation (Equation 3), shown by blue symbols in Figure 2a, reveal that the amplitude of oscillations reach π at the end of the velocity-matching step. This causes a premature switching out of the resonance before reaching the resonant frequency. It is somewhat miraculous that the agreement with the perturbative solution (red line in Figure 2a) is so good. Apparently, it works remarkably well far beyond the range of its formal applicability, |*g_n_*| ≪ 1.

A general single-mode solution for an arbitrary amplitude was obtained by Kulik [30]. The amplitude at the resonance, 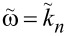, is given by the first solution of the implicit equation [31],


[29]

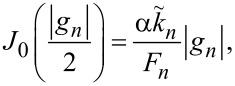



where *J*_0_ is the zeroth-order Bessel function. This equation can be easily solved numerically. It is also possible to obtain an approximate analytic solution by expanding *J*_0_(*x*) ≃ 1 − *x*^2^/4 for small *x*. With such expansion, Equation 29 is reduced to a quadratic equation with the solution


[30]

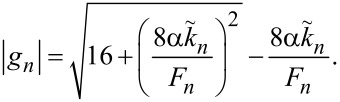



For overdamped JJs, α ≫ 1, it reduces to the small-amplitude result of Equation 25, |*g_n_*| = 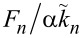. For underdamped JJs, it qualitatively correctly predicts saturation of the amplitude for α→0, although at a value of 4 instead of π. Thus, Equation 30 provides a simple and sufficiently good approximation for a significantly broader range of damping parameters than Equation 25.

### Input resistance

For the practically most important velocity matching mode, *k_n_* = *k*, from Equations 19–21 it follows, *B_n_* = 1, *C_n_* = 0, *F_n_* = 1, leading to a remarkably simple result,


[31]

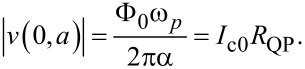



This equation has a straightforward meaning illustrated by the equivalent circuit in Figure 1c. A JJ is a source of a spatially distributed oscillating current, *J_z_* = *J*_c0_sin(ω*t* + *kx*), with a fixed amplitude, *J*_c0_, but spatially dependent phase, *kx*. It couples to the cavity mode via some effective input impedance *Z*_in_. *Z*_in_ depends on ω, *k_n_* and *k* and is, in general, complex. However, since the phase of the current wave is strongly varying along the junction, it is hard to define the phase shift between current and voltage. Therefore, in what follows, I will be talking about the input resistance, *R*_in_ = |*Z*_in_|, defined via the relation


[32]

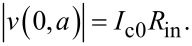



From Equation 26 it follows,


[33]

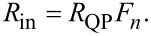



**Figure 3:**
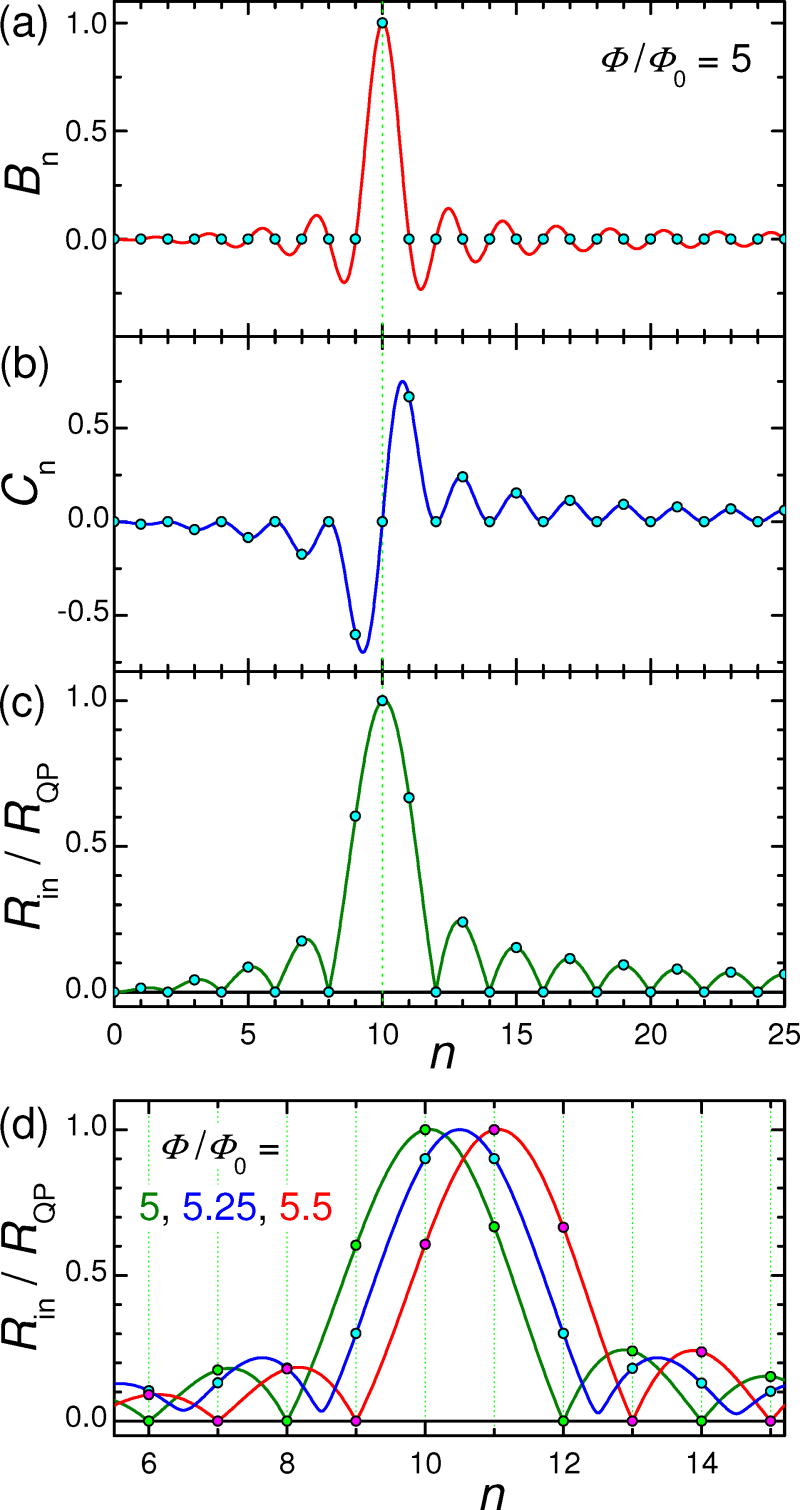
Panels (a) and (b) show mode-number dependence of coefficients *B_n_* and *C_n_*, given by Equation 20 and Equation 21, for the case from Figure 2 with Φ/Φ_0_ = 5. Panel (c) shows the corresponding oscillatory dependence of the input resistance, Equation 28 and Equation 33. (d) Input resistance for Φ/Φ_0_ = 5 (olive), 5.25 (blue) and 5.5 (red). The large *R*_in_ enables good coupling of the cavity mode to the Josephson current.

Figure 3a–c shows, respectively, *B_n_*, *C_n_*, and *R*_in_/*R*_QP_ = *F_n_* versus *n* for the case from Figure 2. Lines are obtained for continuous variation of *n* in Equation 20 and Equation 21, and circles represent the actual cavity modes with integer *n*. From Figure 3c, it is seen that *R*_in_ has a distinct maximum at the velocity matching condition *n* = 2Φ/Φ_0_ = 10. At this point, 
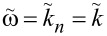
, the wave numbers of the cavity mode and the current wave coincide, leading to a perfect coupling along the whole length of the JJ. Therefore, *R*_in_ = *R*_QP_ and *v* = *I*_c0_*R*_QP_. For other modes, *k_n_* ≠ *k*, the coupling with Josephson current oscillations is much weaker. As seen from Figure 3c, it is oscillating with *n*. For the particular case with integer Φ/Φ_0_, *R*_in_ vanishes for all even modes. This leads to the absence of corresponding Fiske steps in Figure 2a.

The coupling of a cavity mode to the current wave in the JJ depends on magnetic field and flux in the JJ (via the parameter *k*). This is illustrated in Figure 3d for Φ/Φ_0_ = 5 (olive line, the same as in Figure 3c), 5.25 (blue), and 5.5 (red). Although the oscillatory behavior of Fiske step amplitudes is well known [16,29,31], the interpretation of such behavior in terms of the input resistance makes a clear connection to the analysis of patch antennas, for which *R*_in_ is one of the most important parameters. From this point of view, geometrical resonances with large voltage amplitudes appear only for modes coupled to the current source (Josephson oscillations) via a large input resistance, Equation 32. As seen from Figure 3d, the best coupling with maximum, *R*_in_ = *R*_QP_, occurs for the velocity-matching step, *n* = 2Φ/Φ_0_. Modes with *R*_in_ = 0 are not coupled to Josephson oscillations and, therefore, are not excited at all. In particular, there is no coupling to any mode in the absence of an applied field, *R*_in_(*H* = 0) = 0. This is why Fiske steps do not appear at zero field.

### Inclusion of radiative losses in a cavity mode analysis

Finally, in order to calculate radiative characteristics, we need to take into consideration radiative losses. In the previous section, only QP losses in a pure cavity eigenmode were considered. Yet, pure eigenmodes, *E_n_* ∝ cos(*k_n_x*), *H_n_* ∝ sin(*k_n_x*), do not emit any radiation because they do not produce ac magnetic fields at the edges *H_n_*(0,*L*) = 0 [36]. Consequently, the Pointing vector is zero. In other words, eigenmodes have infinite radiative impedance, *Z*_rad_(0,*L*) = *E*(0,*L*)/*H*(0,*L*) = ∞. Therefore, despite large electric fields, the radiated power *P*_rad_ ∝ *E*^2^/*Z*_rad_ is zero [10].

Radiative losses can be included using the equivalent circuit sketched in Figure 1c. Voltage oscillations at the JJ edges are produced by the oscillating supercurrent via the input resistance, Equation 32. The generated electromagnetic power is distributed between internal losses, characterized by the dissipative resistance, *R*_dis_, and radiative losses to free space, characterized by the radiative resistance *R*_rad_. They are connected by the transmission line impedance,


[34]

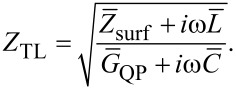



Here *Z*_surf_ is the surface impedance of the electrodes, *G*_QP_ = 1/*R*_QP_ is the quasiparticle conductance, *L* is the inductance, and *C* is the capacitance of the JJ. The bars indicate that the quantities are taken per unit length. For not very high frequencies and temperatures, the surface resistance of Nb electrodes is small (as will be discussed below). For tunnel JJs, *G*_QP_ is also small. In this case,


[35]

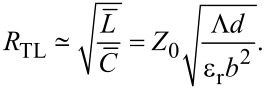



It is very small because *b* ≫ Λ ≫ *d* and can be neglected for all practical cases. Therefore, in Figure 1c we may consider that the dissipative and radiative resistances are connected in parallel. Analysis of patch antennas [36] and numerical calculations for JJs with radiative boundary conditions [10] show that radiative losses can be simply included in the cavity mode analysis by introducing the total quality factor, *Q*_tot_, of the cavity mode with parallel dissipative and radiative channels,


[36]

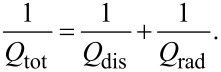



Here, *Q*_dis_ is associated with all possible dissipative losses, such as QP resistance in the JJ as well as surface resistance in electrodes and dielectric losses while *Q*_rad_ represents radiative losses,


[37]

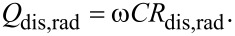



Using definitions of α and *Q*, we can introduce a total damping factor


[38]

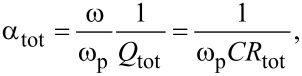



where the total resistance is


[39]

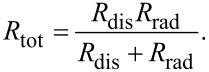



Thus, to include radiative losses, α and *R*_QP_ in the equations above should be replaced by α_tot_ and *R*_tot_. For the *n*-th cavity mode resonance we obtain,


[40]

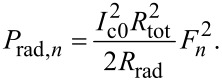



For the most important velocity matching resonance from Equation 31, we obtain


[41]

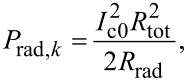



with *R*_rad_ and *R*_tot_ defined in Equation 12 and Equation 39.

### Power efficiency

The total power dissipated in a JJ is given by the product of dc voltage and dc current,


[42]

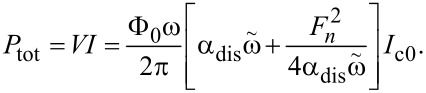



Here, the left factor is the dc voltage, and the right one is the total dc current. It contains the QP current (first term) and the rectified excess current, Δ*I*, (second term). The latter is written using Equation 27 at the resonance condition 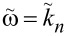. It is important to note that the nonlinear rectification occurs only inside the JJ. Therefore, the damping parameter α_dis_ within the JJ is used for both terms. The first term in Equation 42 describes dissipative dc losses, which generate only heat, *P*_heat_ = *V*^2^/2*R*_dis_. The second term in Equation 42 describes the total power consumed by the cavity mode, *P*_cav_ = *V*Δ*I*. Only this term is participating in radiation. From Equation 39 and Equation 40, we obtain a well-known connection between the radiated power and the power consumed solely by the cavity mode,


[43]

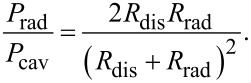



As usual, the maximum emission power is achieved at the matching condition *R*_rad_ = *R*_dis_. In this case, exactly one half of the cavity mode power is emitted and another half is dissipated. This is typical for antennas [36] and is consistent with direct simulations for JJs with radiative boundary conditions [10]. Yet, the overall power efficiency is reduced by the “leakage” QP current in Equation 42, which just produces heat. For the *I*–*V* curves in Figure 2a, the ohmic QP current is more than twice Δ*I* at the velocity matching step. Therefore, the total power efficiency, *P*_rad_/*P*_tot_, for such moderately underdamped JJ will not exceed 50/3 ≃ 17%. Since the leakage current decreases with increasing *R*_QP_, strongly underdamped JJs are necessary for reaching a power efficiency of approx. 50%. This is the case for Nb tunnel JJs [9] and for high-quality intrinsic JJs in Bi-2212 high-*T*_c_ cuprates, for which the quality factor may exceed several hundreds [32] and Δ*I* can be several times larger than the leakage QP current [9,32].

## Discussion

### Estimation of parameters

Let us estimate characteristic impedances for the case of Nb/AlO*_x_*/Nb tunnel JJs, which are used in state-of-the-art FFOs [9,11]. I assume that *a* = 100 μm, *b* = 10 μm, *d* = 2 nm, ε_r_ = 10, *d*_1_ = *d*_2_ = 100 nm, the zero-temperature London penetration depth λ_L0_ = 100 nm, *J*_c0_ = 5 × 10^3^ (A/cm^2^), *I*_c0_ = *J*_c0_*ab* = 50 mA, and the characteristic voltage *I*_c0_*R_n_* = 1 mV. This yields, *R_n_* = 20 mΩ, *C* = 44.25 pF, Λ = 272.6 nm, inductance L^*^ = μ_0_Λ*a*/*b* = 3.43 pH, and *c*_0_/*c* = 2.71 × 10^−2^.

#### Surface resistance

Within the two-fluid model, the surface resistance of two superconducting electrodes can be written as [41]:


[44]

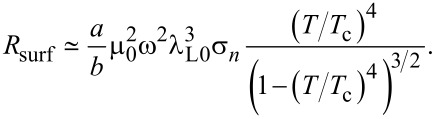



Here, σ*_n_* is the normal state conductivity. This approximation is valid for not very high temperatures, *T*/*T*_c_ *<* 0.8. Using typical parameters for sputtered Nb films, σ*_n_* ≃ 1.75 × 10^5^ (Ω·cm)^−1^ [42], frequency *f* = 400 GHz, and *T*/*T*_c_ = 0.5, we obtain: *R*_surf_ ≃ 0.12 Ω.

#### Transmission line impedance

The TL impedance is given by Equation 34 where *G*_QP_ = 1/*R*_QP_. For tunnel JJs, *R*_QP_ ≫ *R_n_* at sub-gap voltages. I will assume *R*_QP_ = 25*R_n_*, typical for Nb tunnel JJs [9,11]. This gives *R*_QP_ = 0.5 Ω and *G*_QP_ = 2 Ω^−1^. At *f* = 400 GHz, ωL^*^ = 8.61 Ω, ω*C* = 111.2 Ω^−1^, and *Z*_TL_ ≃ 0.278 + *i*0.0015 Ω. It practically coincides with the resistance of an ideal TL, Equation 35. The value of *Z*_TL_ is only slightly affected by an ill-defined QP resistance and remains practically the same even if we use the upper limit, *G*_QP_ = 1/*R_n_*. Importantly, *Z*_TL_ is small because of very small *d*.

#### Dissipative resistance

The effective dissipative resistance is affected by all sources of dissipation, including QP and dielectric losses in the junction barrier and surface resistance in electrodes. According to Equation 37, *R*_dis_ is defined via the effective quality factor, *Q*_dis_, which can be written as:


[45]

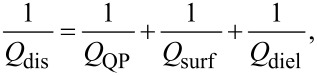



where *Q*_QP_, *Q*_surf_ and *Q*_diel_ are determined by QP, surface, and dielectric losses, respectively. QP and surface resistance contribution can be accounted for using the TL analysis. The quality factor of a TL is determined by the relation




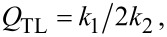




where *k*_1_ and *k*_2_ are real and imaginary parts of the wave number in the TL, *k* = *k*_1_ − *ik*_2_. They are obtained from the TL dispersion relation,




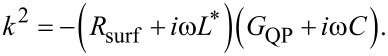




Taking into account that *G*_QP_ = 1/*R*_QP_ ≪ ω*C*, *R*_surf_ ≪ ωL^*^, and 
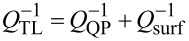
, we obtain


[46]

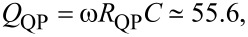




[47]

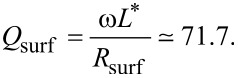



Dielectric losses in the AlO*_x_* barrier of a JJ were estimated in [43]. At *f* ≃ 10 GHz, *Q*_diel_ ≈ 10^4^. Although it should decrease at *f* = 400 GHz, we anticipate that it is still in the range of ca. 10^3^. Therefore, dielectric losses are negligible, compared to QP and surface loses. Assuming *Q*_diel_ = 500, we obtain, from Equations 45–47, *Q*_dis_ = 29.48 and *R*_dis_ ≃ 0.265 Ω. It is close to the effective dissipative resistance of the TL,


[48]

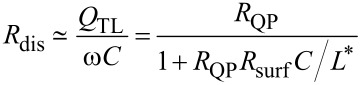



#### Radiative and total resistances

From Equation 12 and Equation 8, taking into account the smallness of *c*_0_/*c*, we can write,


[49]

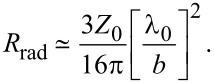



Substituting λ_0_ = 750 μm for *f* = 400 GHz, we obtain a very large value, *R*_rad_ ≃ 126.5 kΩ. Since *R*_rad_ ≫ *R*_dis_, the total resistance, Equation 39, is *R*_tot_ = 0.265 Ω ≃ *R*_dis_.

Table 1 summarizes characteristic resistances.

**Table 1:** Estimation of characteristic resistances (in ohms) for a Nb/AlO*_x_*/Nb tunnel junction with sizes *a* = 100 μm, *b* = 10 μm, *d* = 2 nm, *d*_1_ = *d*_2_ = 100 nm, *J*_c0_ = 5000 (A/cm^2^), at *T*/*T*_c_ = 0.5 and *f* = 400 GHz.

*R* *_n_*	*R*_QP_	*R*_surf_	*R*_TL_	ωL^*^	(ω*C*)^−1^	*R*_dis_	*R*_rad_	*R*_tot_

0.02	0.5	0.12	0.28	8.6	0.009	0.265	126.5k	0.265

#### Radiation power

From Equation 41, we get the maximum radiation power at the velocity matching condition, *P*_rad_*_,k_* ≃ 0.7 nW. It is much smaller than the total dc power at the velocity matching step, approx. Φ_0_*fI*_c0_ ≃ 40 μW. The corresponding power efficiency of approx. 10^−5^ reflects the key problem for using FFO as a free-space oscillator.

### Whom to blame?

The very low radiation power efficiency of a JJ is colloquially attributed to “impedance mismatch”. However, so far, there was no clear understanding of what mismatches with what. A long-living misconception is that the mismatch is between the TL and free-space impedances, *Z*_TL_ ≪ *Z*_0_ [16]. However, this is not the source of the poor performance. On the contrary, it is beneficial to have a small TL impedance, connecting two radiating slots in a patch antenna [36]. The small *Z*_TL_ does not affect antenna performance and can be neglected.

The real source of the problem becomes apparent from Equation 41. It is associated with the more than five orders of magnitude mismatch between the total and radiative resistances, *R*_tot_ ≪ *R*_rad_, see Table 1. There are two main reasons for the mismatch: (i) The smallness of the junction width with respect to the free-space wavelength. The factor (λ_0_/*b*)^2^ in Equation 12 and Equation 49 leads to a very large *R*_rad_ ≫ *Z*_0_. (ii) The smallness of the junction resistance, *R*_QP_ ≪ *Z*_0_. The huge mismatch indicates that a JJ alone does not work as a free-space oscillator.

### What to do?

Accurate matching between radiative and junction resistances is necessary for efficient emission into free space. Therefore, *R*_QP_ should be increased and *R*_rad_ decreased to a fraction of *Z*_0_. However, this is not possible for the standard FFO geometry as sketched in Figure 1a. Indeed, increasing *R*_QP_ would require the reduction of junction sizes, which would lead to even faster increase of *R*_rad_. Alternatively, *R*_QP_ can be increased by decreasing *J*_c0_, but this will not reduce *R*_rad_. Therefore, the impedance matching requires modification of the oscillator geometry.

There are many ways of coupling a Josephson oscillator to free space. First, I note that biasing electrodes that are attached to the junction, significantly affect the net impedance. Since the total length of the electrodes (few millimeters) is larger than λ_0_, the electrodes will reduce the net impedance and, thus, improve impedance matching with free space [17]. Analysis of large JJ arrays demonstrated that long electrodes may act as a traveling wave antenna, facilitating a power efficiency of several percent at *f* = 0.1–0.2 THz [44–45], which is much better than approx. 10^−5^ estimated above for the bare junction without electrodes. In [11], a free-space oscillator based on an FFO, coupled to a double-slot antenna, was demonstrated. Although the power efficiency was not specified, a detected off-chip signal up to 55 dB higher than the background noise was reported at *f* = 0.5 THz. In [27], a mesa structure containing several hundreds of stacked Bi_2_Sr_2_CaCu_2_O_8+δ_ intrinsic JJs was implemented in a turnstile antenna. A radiation power efficiency up to 12% at *f* ≃ 4 THz was reported. The record high efficiency was attributed to a good impedance matching with free space [17]. In [24], a Bi_2_Sr_2_CaCu_2_O_8+δ_ mesa was implemented into a patch antenna and far-field emission at *f* = 1.5 THz was reported.

Common for all mentioned approaches is that the junctions, which are small compared to λ_0_ and, according to Equation 49, have poor coupling to free space, are coupled to large passive elements, comparable with λ_0_. These elements act as microwave antennas, enabling good impedance matching and enhancing the power efficiency for emission in free space. The target parameters for such oscillators are *f* ≈ 1–10 THz, a high power-efficiency of approx. 50% and a sufficiently high off-cryostat power above 1 mW.

#### Josephson patch oscillator

**Figure 4:**
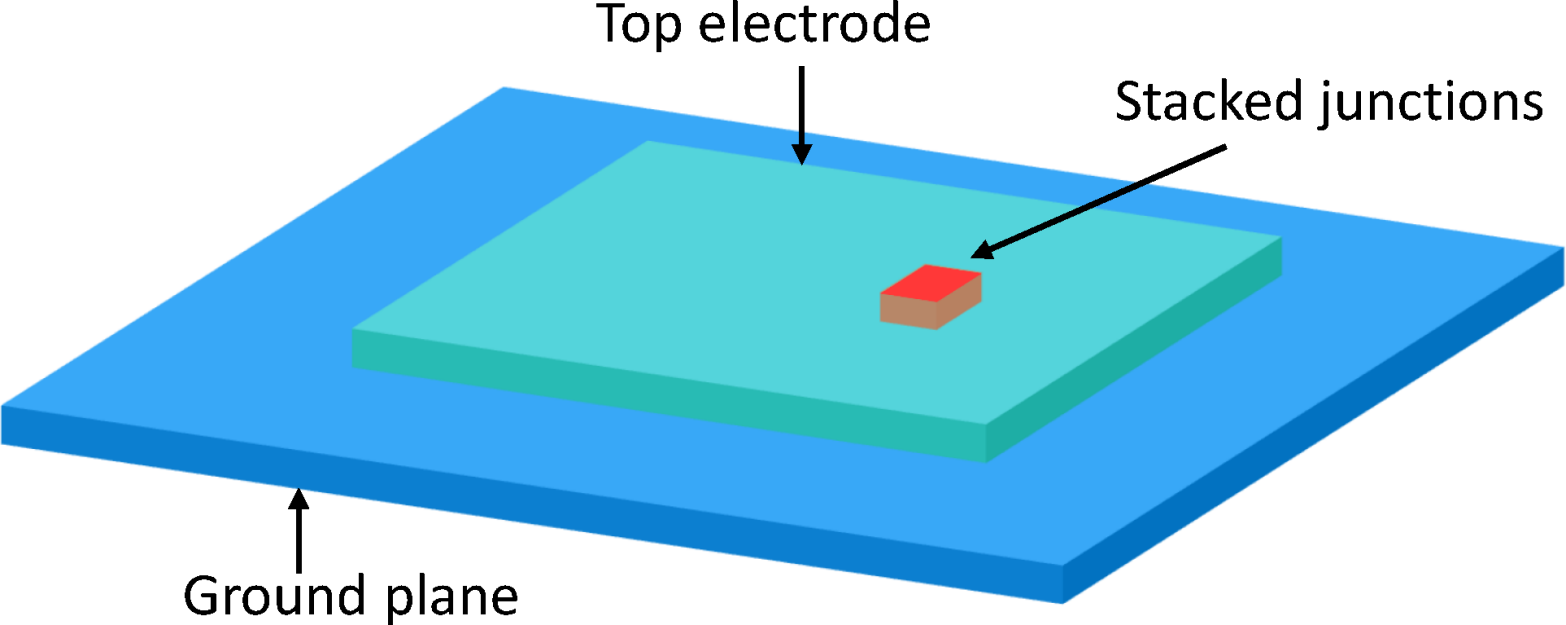
A proposed design of the impedance-matched free-space Josephson oscillator. Here, a small stack of Josephson junctions (red) is sandwiched between two large superconducting electrodes, namely the ground plane (blue) and the top electrode (light blue). The stack is acting as a source of microwave current (feed-in) for the patch antenna formed by the electrodes.

Since in this work I consider patch antennas, below I will dwell on the patch antenna approach, discussed by Ono and co-workers [24]. Figure 4 shows a design of a Josephson patch oscillator (JPO). Here, small junctions (red) are acting as an excitation source for a superconducting patch antenna. The bottom junction electrode (blue) forms the ground plane, and the top electrode (cyan) creates the patch antenna with sizes (*a*, *b*), comparable to λ_0_. In principle, the JPO can be driven by a single JJ. However, as follows from the estimation above (see Table 1), raising the junction resistance to the desired *Z*_0_ level would require a drastic (100 times) reduction of the junction area. This will also lead to a proportional reduction of *I*_c0_ and the net available power. Therefore, a better strategy is to use a stack of JJs with large-enough area, enabling high-enough *I*_c0_. The number of JJs, *N*, is an additional controllable parameter, allowing for fine-tuning of *R*_n_ and *R*_tot_. Furthermore, in-phase synchronization of *N* JJs would provide the *N*-fold increment of the oscillating voltage *v*(0,*L*), leading to a superradiant amplification of the emission power, *P*_rad_ ∝ *N*^2^ [10].

Moderate-size (approx. 10 μm) Bi_2_Sr_2_CaCu_2_O_8+δ_ mesa structures are optimal for JPOs. The *R_n_* of such mesas can be easily raised to several hundred ohms, while maintaining *I*_c0_ of a few milliamperes. This facilitates the optimal net power level ≈*I*^2^*R_n_* of several milliwatts [24,27]. It is small enough for obviation of catastrophic self-heating, which is one of the major limiting factors for superconducting devices [17,27]. Simultaneously, it is large enough to enable emission above 1 mW, provided the radiation power efficiency is close to the optimal approx. 50%.

The operation frequency should be aligned with the Josephson frequency at the characteristics voltage, *I*_c0_*R*_n_, of JJs. For operation at the primary 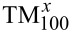 mode, one side of the patch should be *a* ≃ λ/2, where λ = λ_0_/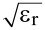 is the wavelength inside the patch and ε_r_ is the relative dielectric permittivity of the insulation layer between the patch electrodes. The other size, *b*, is adjustable and strongly affects the patch antenna performance. For *b* ≪ λ_0_, the radiative conductance per slot is given by Equation 5. In the opposite limit, it becomes [36]


[50]

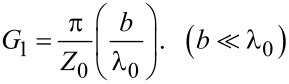



One of the most important parameters of the emitting antenna is the directivity, *D*, of the radiation pattern. A rectangular patch at the 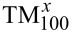 mode has the main lobe directed perpendicular to the patch (in the *z*-axis direction) with [36]




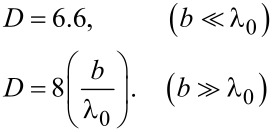




A good free-space emitter should have a value for *D* as large as possible. From this point of view, it is preferable to have fairly wide antennas *b* ∼ λ_0_.

Finally, the position (*x*, *y*) of the stack plays an important role in the selection of the excited cavity mode. To excite solely the 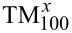 mode, the stack should be placed at *x* close to one of the radiating slots, that is, *x* ∼ *a* and *y* = *b*/2. The position *x* of the stack affects the effective input resistance of the antenna and provides another adjustable parameter for patch antenna operation, along with the shape of the top electrode [35–36,46]. The FFO input resistance, Equation 33, is not relevant for JPOs, because it describes coupling to an internal cavity mode within the JJ. In JPOs, the Josephson current is coupled to an external cavity mode in the patch. Since the patch is much larger than the JJ, the feed-in of the JPO is not distributed (in contrast to a FFO). Consequently, there is no need for a magnetic field. The best coupling occurs at *H* = 0, corresponding to the homogeneous distribution of the Josephson current. Generally, operation of JPOs is described by the standard patch antenna theory [36]. The only interesting physics is associated with synchronization of JJs in the stack [10], which can be forced by the high-quality cavity mode in the antenna [47].

## Conclusion

I described a distributed, active patch antenna model of a Josephson oscillator. It expands the standard transmission line model of a patch antenna, taking into account the spatial-temporal distribution of the input Josephson current density in a Josephson junction. In the presence of a magnetic field and fluxons, the distribution of the oscillatory component of current is nonuniform. This nonuniformity is essential for operation of a Josephson flux-flow oscillator and determines the effective input resistance, which enables the coupling between the Josephson current and the cavity modes in the junction. The presented model allows for the explicit application of many patch antenna results and facilitates full characterization of the device, including emission power, directivity, and power efficiency. The model explains the low power efficiency for emission in free space. It is primarily caused by the smallness of the junction width compared to the free-space wavelength and the corresponding mismatch between very large radiative and small junction resistances. The model clarifies which parameters can be changed to improve FFO characteristics. Finally, I discussed the design of a Josephson patch oscillator that can reach high power for emission in free space with the optimal power efficiency of approx. 50%.

## Appendix

Definition of variables (Table 2).

**Table 2:** Definition of variables.

Variable	Definition	Properties

*a*, *b*	junction length and width in (*x*, *y*) plane	*a* ≫ λ_J_, *b* ∼ λ_J_
α	quasiparticle damping factor	α = 1/ω_p_*R*_QP_*C* = 1/*Q*_QP_(ω_p_)
*C*	junction capacitance	*C* = ε_0_ε_r_*ab*/*d*
*c*_0_	Swihart velocity	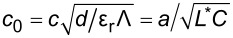
*d*, *d*_1_*_,_*_2_	thicknesses of JJ interlayer and the two electrodes	*d* ≪ *b* ≪ *a*
Φ	flux in the junction	Φ = *H_y_*Λ^*^a
Φ_0_	flux quantum	Φ_0_ = *h*/2*e*
*J*_c0_, *I*_c0_	maximum critical current density and critical current	*I*_c0_ = *J*_c0_*ab*
*k*	field-induced phase gradient	*k* = 2πΦ/Φ_0_*a*
*k* *_n_*	wave number of a cavity mode	*k_n_* = (π/*a*)*n*
L^*^, *L*_□_	inductance of JJ and inductance per square	L^*^ = μ_0_Λ*a*/*b*, *L*_□_ = μ_0_Λ
λ_L1,2_	London penetration depths of the two JJ electrodes	–
λ_0_	wavelength in free space	–
λ	wavelength in the patch antenna	λ = λ_0_/ 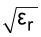
λ_J_	Josephson penetration depth	λ_J_ = [Φ_0_/2πμ_0_*J*_c0_Λ]^1/2^ = *c*_0_/ω_p_
Λ	characteristic length associated with JJ inductance	Λ = *d* + λ_L1_coth(*d*_1_/λ_L1_) + λ_L2_coth(*d*_2_/λ_L2_)
Λ^*^	effective magnetic thickness of the JJ	Λ^*^ = *d* + λ_L1_tanh(*d*_1_/2λ_L1_) + λ_L2_tanh(*d*_2_/2λ_L2_)
η	Josephson phase difference	–
ω_p_	Josephson plasma frequency	ω_p_ = [2π*I*_c0_/Φ_0_*C*]^1/2^
ω_J_	angular Josephson frequency	ω_J_ = ∂η/∂*t* = 2π*V*_dc_/Φ_0_
ω*_n_*	cavity mode angular frequency	ω*_n_* = *c*_0_*k_n_*
*R*_QP_, (*r*_QP_)	subgap quasiparticle resistance, (per unit area)	*r*_QP_ = *R*_QP_*ab*
*R*_dis_	net dissipative resistance	–
*R*_surf_	surface resistance of electrodes	–
*R*_n_	normal state resistance of the JJ	–
*R*_TL_	transmission line resistance	–
*R*_rad_	radiative resistance	–
*R*_in_	effective input resistance of the JJ	–
*R*_tot_	total load resistance of the JJ	–
